# Saliva-based genomic signatures for early detection of oral squamous cell carcinoma: the state and perspectives of translation

**DOI:** 10.3389/fgene.2026.1831378

**Published:** 2026-10-09

**Authors:** Hiren Hansraj Patadiya, Satya Upadhyayula, Vrunda T. Adeshara

**Affiliations:** 1 Boston University, Boston, MA, United States; 2 Private Practice, Watertown, MA, United States; 3 Comprehensive Community Action Plan, Cranston, RI, United States

**Keywords:** DNA methylation, early diagnosis, extracellular vesicles, genomic biomarkers, liquid biopsy, microRNA, next-generation sequencing, oral potentially malignant disorders

## Abstract

Oral squamous cell carcinoma (OSCC) is one of the leading causes of cancer morbidity and mortality globally, and prognosis is closely related to the stage at diagnosis. Although advances in surgery, radiotherapy, and systemic therapies have improved outcomes, late presentation remains a major limitation to survivability. Saliva has emerged as a promising noninvasive diagnostic biofluid due to its proximity to tumors, ease of repeated collection, and the presence of tumor-derived nucleic acids. From 2020 to 2024, research increasingly focused on integrated genomic signatures combining somatic mutations, DNA methylation patterns, and RNA-based biomarkers (including mRNA, microRNA, and other noncoding RNAs) in whole saliva, cell-free fractions, and extracellular vesicles. Technological advancements such as digital polymerase chain reaction (PCR), targeted next-generation sequencing, and computational modeling have enhanced sensitivity for detecting low-abundance tumor signals and enabled longitudinal monitoring strategies. However, translation into routine clinical practice remains limited because of pre-analytical heterogeneity, inflammatory confounding, batch effects, late-stage enriched cohorts, and lack of large prospective multicenter validation. This review synthesizes current evidence, evaluates analytical and clinical readiness, and proposes a translational roadmap for clinical integration.

## 1 Introduction

Oral squamous cell carcinoma (OSCC) is the most prevalent malignancy of the oral cavity and a significant constituent of head and neck squamous cell carcinomas (HNSCCs). The occurrence of tobacco use, alcohol use, exposure to betel quid, human papillomavirus patterns of prevalence across disparate subsites, and disparities in healthcare access continue to reflect significant geographic disparity in incidence and mortality of the disease under global surveillance. The reason behind the importance of earlier diagnosis is that the stage at which the cancer is diagnosed is closely linked to survival and functional outcomes: at an early stage of OSCC, often less extensive resection and higher life quality are possible, but at the advanced stage, multimodal therapy with high morbidity is unavoidable (Warnakulasuriya, 2009; Bray et al., 2021).

Clinical diagnosis depends on oral examination followed by biopsy. Histopathology is the reference standard, but it is invasive, might not reflect spatial heterogeneity, and is not suitable for either repetitive monitoring in high-risk individuals or longitudinal surveillance of oral potentially malignant disorders (OPMDs). Lesion identification can be aided by diagnostic adjuncts such as vital stains, autofluorescence, brush cytology, and optical imaging, which vary in performance among operators and devices and do not directly measure molecular evolution (Lingen et al., 2008).

The purpose of liquid biopsy is to identify tumor-related biomarkers in the available fluids. Saliva is particularly attractive for OSCC because the salivary environment has direct access to the tumor; the tumor proximity can increase local biomarker concentration compared to blood in local disease, particularly in the early stages. Saliva collection is noninvasive, repeatable, inexpensive, and can be used in conjunction with dental-chair or community screening processes. A growing body of evidence shows that saliva-based molecular signatures are being used:

(i). To triage concerning suspicious lesions;
(ii). To monitor relapse and minimal residual disease following definitive therapy; and
(iii). To possibly detect the disease early in high-risk groups (Papale et al., 2022; Nassar et al., 2024; Yang et al., 2024).

A genomic signature (described in this review) is a risk signal that is made up of a combination of nucleic acid-based signals. Clinically deployable tests are likely to examine many markers (e.g., methylation and miRNA) as opposed to a single marker because the oral cavity is very inflammatory and rich in microbes. We focus on evidence from 2020 to 2024 and explain it through the prism of analytical validity, clinical validity, and implementation based on the intended use.

Salivary nucleic acid signatures are increasingly acknowledged not only in oral tumors but also in many malignancies, indicating their extensive utility as liquid biopsy techniques. These encompass circulating tumor DNA, microRNAs (miRNAs), long noncoding RNAs (lncRNAs), and extracellular vesicle (EV)-associated nucleic acids, which collectively elucidate tumor biology and disease progression (Papale et al., 2022; Nassar et al., 2024). Because they are noninvasive and can detect real-time changes in molecules, they are especially useful for finding diseases early, determining who is at risk, and keeping an eye on diseases over time.

## 2 Materials and methods

This is a literature review submitted to Frontiers as a narrative review. We selected literature in 2020–2024 on saliva-based genomic, epigenomic, and EV biomarkers of OSCC. When evidence on OSCC-specific studies for a specific assay strategy was limited, we added related evidence on HNSCC when it directly informed applications in the mouth cavity. Earlier pre-2020 work was not the main evidence base but was selected selectively to put biomarker classes into context. Evidence was structured according to the presence of a translational pathway: (i) biological plausibility and selection of matrices; (ii) analytical validity (sensitivity, reproducibility, and QC); (iii) clinical validity in prespecified intended uses (screening, triage, and surveillance); and (iv) clinical utility, feasibility, and equity-conscious implementation.

## 3 Saliva-based genomic detection is biologically based

Saliva can also be used as a tumor-adjacent fluid biopsy (Section 3.1).

Major and minor salivary glands produce saliva, which is combined with desquamated cellular material and gingival crevicular fluid and mixed. It has soluble proteins and metabolites, microbial products, EVs, and nucleic acids. In oral cavity tumors, lesions are in direct contact with saliva; biomolecules may get into saliva by passive shedding, micro-ulceration and bleeding, and active secretion. This closeness justifies the fact that saliva could provide a better local tumor signal than blood for tumors that are limited to the oral cavity, particularly at earlier tumor stages (Papale et al., 2022; Khijmatgar et al., 2023).

Two implications for practice ensue. First, sequential observation is possible with repeated collection and within-person trend analysis, which can be of great use in surveillance and in the follow-up study of OPMD. Second, the salivary molecular milieu may be overwhelmed by local oral conditions (periodontitis, mucositis, and infection), so signatures must be confounder-aware.

### 3.1 The location of tumor signals: whole saliva, cell-free fraction, and EVs

Salivary nucleic acid can be recorded in various fractions. Whole saliva consists of cellular and fluidic elements. The cellular pellet is a cellular-enriched sample with exfoliated epithelial cells and leukocytes; it may have tumor DNA but may be filled with inflammation. The cell-free supernatant includes the released cell-associated and vesicle-associated components with cells and their RNAs. EVs are membrane-bound particles released by cells that carry proteins, lipids, DNA, mRNA, miRNAs, and other noncoding RNAs. Exosomes are one subtype of EVs and have been the most extensively investigated in saliva-based diagnostics because they protect nucleic acids from degradation and may enrich tumor-derived molecular cargo (Salivary exosomes as biomarkers for, 2023).

Fractionation will make it more complicated, so near-term implementation will probably entail either standard whole-saliva workflows with stabilization or partial fractionation that could be done in a regular clinical lab. Specialist centers can implement EV enrichment for high-sensitivity surveillance or mechanistic profiling, whereas primary care triage would likely prefer the simpler workflow.

### 3.2 Confounding through inflammation, microbiome changes, and field cancerization

The mouth is a very special testing environment as inflammatory disease is prevalent and may cause biomarker signal overlap with cancer-related pathways. Host DNA/RNA and inflammatory vesicles are released in periodontitis and gingivitis and change the microbiome. Additional layers of host and microbial variation are brought about by tobacco, alcohol, and betel quid exposures. Moreover, cancerization of the mucosa that occurs chronically in the field may produce molecular changes that are above the tumor, which may be beneficial in risk stratification but make interpretation of the lesion more difficult. Biomarkers should thus be checked against clinically realistic control groups, such as inflammatory and OPMD disorders.

This may be achieved in practice by gathering systematic oral health metadata, such as periodontal condition and oral hygiene activity; inflammatory control recruitment; and mathematical models that isolate malignant signatures of inflammatory action. The failure to do so is among the primary causes of failure of case–control biomarkers to translate.

Collection method is one source of variability. Unstimulated whole saliva as such indicates basal secretion; however, it differs in viscosity and volume. The stimulated saliva is larger, although it may be diluted. Oral rinse/gargle samples have decreased viscosity and improved handling, yet they are vulnerable to volume, length, and compliance. The preparation of the patient must be standardized (no eating, no drinking, no smoking, and no brushing during a certain period of time). Any blood contamination should be recorded, as it may bring circulating nucleic acids and may confound local tumor indicators.

In the case of translation, the simplicity of the protocol is essential: dental clinics need a minimum number of steps; oncology surveillance can be more complicated. Research must indicate what was carried out in a protocol (during what time of the day, through what device, and what volume was delivered) to make it reproducible.

### 3.3 Processing, stabilization, and storage

Nucleases and high concentrations of bacteria are present in saliva. Therefore, time-to-processing, stabilization buffers, and cold-chain control have effects on nucleic acid integrity. In the case of cfDNA/cfRNA assays, separation of the supernatant should be immediate so as to minimize cell lysis, which may dilute tumor signal. Stabilization buffers have the potential to minimize degradation; however, they must be confirmed to be compatible with downstream assays.

EV workflows introduce extra variability. Precipitation techniques are simple and can co-isolate contaminants; ultracentrifugation is also good at purifying with reduced equipment but is equipment-heavy; size-exclusion chromatography is also capable of purifying contaminants better with less equipment but may reduce yield. To be implemented, it is likely that either simplified EV workflows are implemented or strong whole-saliva assays that measure a proxy of EV signal will be developed.

### 3.4 Quality control and reporting of reproducibility

Studies must at least indicate the collection mode, processing time, centrifugation conditions, storage conditions, freeze–thaw cycles, extraction mode, and internal controls. STARD-aligned reporting should be employed in the diagnostic accuracy work. In the case of machine learning signatures, feature selection, model training, calibration, and external validation should be transparently reported to avoid exaggerated performance estimates.

## 4 Salivary DNA-based biomarkers

### 4.1 Longitudinal surveillance and detection of somatic mutation

The goal of somatic mutation detection is to detect the tumor-derived salivary DNA or cfDNA variation. The most developed scheme is tumor-informed monitoring: tumor tissue variants are discovered, after which they are followed in saliva. A 2021 OSCC longitudinal study reported the collection of paired preoperative and postoperative saliva and plasma; targeted deep sequencing made feasible the possibility of tracking variants earlier and implied earlier detection of ctDNA in some recurrent cases as opposed to traditional monitoring (Cui et al., 2021). In a pilot study published in *Frontiers Oncology* in 2024, a systematic 52-gene panel was used to identify somatic variants in serial salivary DNA to detect minimal residual disease and relapses in HNSCC (Dal et al., 2024).

In clinical practice, mutation tracking would be appealing for surveillance because specificity is high in tracking patient-specific variants. Among the translational questions, there is the need to define actionable thresholds, discordance of saliva/plasma management, and incorporation of molecular results into imaging and biopsy pathways. Future interventional trials should be conducted to verify that an earlier molecular diagnosis can have a significant clinical impact.

### 4.2 DNA Plasma ctDNA MRD: lessons from head and neck cancer

Lessons of the methodology are offered by plasma ctDNA work. The LIONESS study, published in the *British Journal of Cancer* in 2022, assessed personalized ctDNA analysis of minimal residual disease and recurrence detection in p16-negative HNSCC and showed that tumor-informed assays were feasible and that serial sampling had value (Flach et al., 2022). In 2024, meta-analyses proved the correlation between ctDNA positivity and worse prognosis, the necessity of uniform sampling regimes, and the standard definition of molecular relapse (Yang et al., 2024). These models apply to saliva, and the added need is to control oral inflammatory confounding.

### 4.3 Tumor-naïve DNA tumor triage and screening

Tumor-naïve approaches are required to take saliva DNA testing to the next stage of triage and screening. These can be predefined mutation panels, methylation classifiers, and in-development cfDNA fragmentomics. Fragmentomics finds extensive application in blood-based early detection and uses fragment length and tumor-derived end motifs as tumor-derived signals; fragmentation adaptation to saliva is feasible but immature. In the near term, tumor-naïve DNA methods will probably be adopted as a component of multilayered signatures (e.g., methylation + miRNA + limited DNA variants) to enhance robustness across cohorts.

## 5 Salivary DNA methylation biomarkers

### 5.1 Why methylation has a future

DNA methylation changes occur at an early stage of carcinogenesis and may indicate the presence and effects of the field. The stability of methylation markers is relatively high compared with RNA, which is an advantage in saliva. A review in *Cancers* (2022) addressed cell-free DNA-based methylation patterns in saliva as early predictors of head and neck cancer and demonstrated the technical advances and the necessity of large prospective clinical trials (Birknerova et al., 2022). To a greater extent, the 2024 head and neck liquid biopsy reviews focus on methylation as a central layer of diagnosis and monitoring and place it as a supplementary layer of mutation monitoring (Nassar et al., 2024; Yang et al., 2024).

Methylation signatures could be well applicable to lesion triage and risk stratification in the OPMD pathways because an epigenetic alteration may precede invasion.

### 5.2 Assay methods and analytical issues

Methylation analyses normally involve treatments that entail conversion of bisulfite, then methylation-specific polymerase chain reaction (PCR), or directed sequencing. The variability in analysis may be due to incomplete conversion, low-input DNA, and variation in selection of locus. Conversion controls and internal standards must be added to panels for clinical use. Inflammatory controls should be validated, as oral inflammation can affect methylation patterns.

## 6 Salivary RNA signatures

### 6.1 Signals of transcriptomics (mRNA) and pathway level

Tumor-associated and host-response pathways can be expressed in the salivary mRNA. Recent reviews of salivaomics also highlight that transcriptomics remains valuable because it is included in multi-marker models, although RNA is sensitive to degradation before analysis (Papale et al., 2022). Translation hence relies on standardized stabilization, processing, and perhaps EV enrichment.

A limited qPCR panel with strong ability to contend with variation is the most plausible RNA approach in triage. To improve the stability and specificity of screening applications, RNA might have to be combined with methylation.

### 6.2 MiRNAs: synthesis of evidence and translational implications

MicroRNAs (miRNAs), a class of small noncoding RNAs, are relatively stable because they are frequently enclosed within EVs or protein complexes. A systematic review and meta-analysis not only demonstrated promising diagnostic performance of salivary and blood-derived miRNAs for OSCC but also highlighted substantial heterogeneity across studies (Liu et al., 2021). Likewise, a 2024 meta-analysis supported salivary miRNAs as auxiliary biomarkers while emphasizing the need for standardized methodologies and external validation before routine clinical application (Nguyen and Nonaka, 2024). Overall, miRNA biomarkers remain in the clinical validation stage rather than routine clinical use.

### 6.3 Other noncoding RNAs and integrated RNA models

Other noncoding RNAs, including lncRNAs and circular RNAs (circRNAs), are increasingly implicated in OSCC pathogenesis (Papale et al., 2022; Chandrasekaran et al., 2024). Compared with miRNAs, however, evidence for lncRNAs and circRNAs remains limited and largely exploratory, and these biomarkers have not yet progressed beyond early validation studies (Papale et al., 2022; Salivary exosomes as biomarkers for, 2023). Exosomal nucleic acids may further improve biomarker stability by protecting RNA from degradation. However, variability in isolation methods and lack of standardized protocols continue to limit clinical translation (Salivary exosomes as biomarkers for, 2023). Consequently, these analytes are currently best regarded as components of future integrated multi-omic biomarker panels rather than standalone diagnostic tests (Nassar et al., 2024; Yang et al., 2024).

## 7 Exosome-based biomarkers and EVs

### 7.1 EVs are stabilizing and enriching compartments

Because EVs protect their molecular cargo from enzymatic degradation, they improve the stability of tumor-derived nucleic acids in saliva and may enhance biomarker detection (Salivary exosomes as biomarkers for, 2023). This can be attained especially in saliva, where nucleases and bacteria can break down RNA rapidly. A 2023 study of salivary exosomes in OSCC and OPMDs found that a practical variation in exosome yield among methods of sampling and proposed discriminative behavior to a small panel of exosomes, highlighting the translational significance of a choice of fraction (Salivary exosomes as biomarkers for, 2023).

EV-associated nucleic acids (exosomal miRNAs and other RNAs) can be used to develop a stable detection method, although EV processing complicates the process. Achieving translational advancements will necessitate either simplified EV procedures or the creation of whole-saliva assays that can reliably measure EV-derived signals.

### 7.2 Barriers to standardization and how to remove them

One of the leading sources of variability is the EV isolation method. Precipitation techniques may introduce contaminants. Ultracentrifugation is equipment-intensive, and size-exclusion chromatography can change yield and purity. Isolation technique, vesicle characterization measures, and normalization plan should be reported in studies. Validation will most likely require multicenter participation or centralized processing (Figure 1):

- Patient preparation (fasting and oral restrictions)
- Saliva collection (unstimulated/stimulated/oral rinse)
- Sample processing (centrifugation and fractionation)
- Biomarker extraction (DNA, RNA, and EVs)
- Analytical techniques (PCR, next-generation sequencing (NGS), and methylation assays)
- Data integration (multi-omic modeling and AI)
- Clinical application (screening, triage, and surveillance) (adapted from salivaomics and liquid biopsy frameworks; Papale et al., 2022; Nassar et al., 2024; Nonaka and Wong, 2023).

**FIGURE 1:**
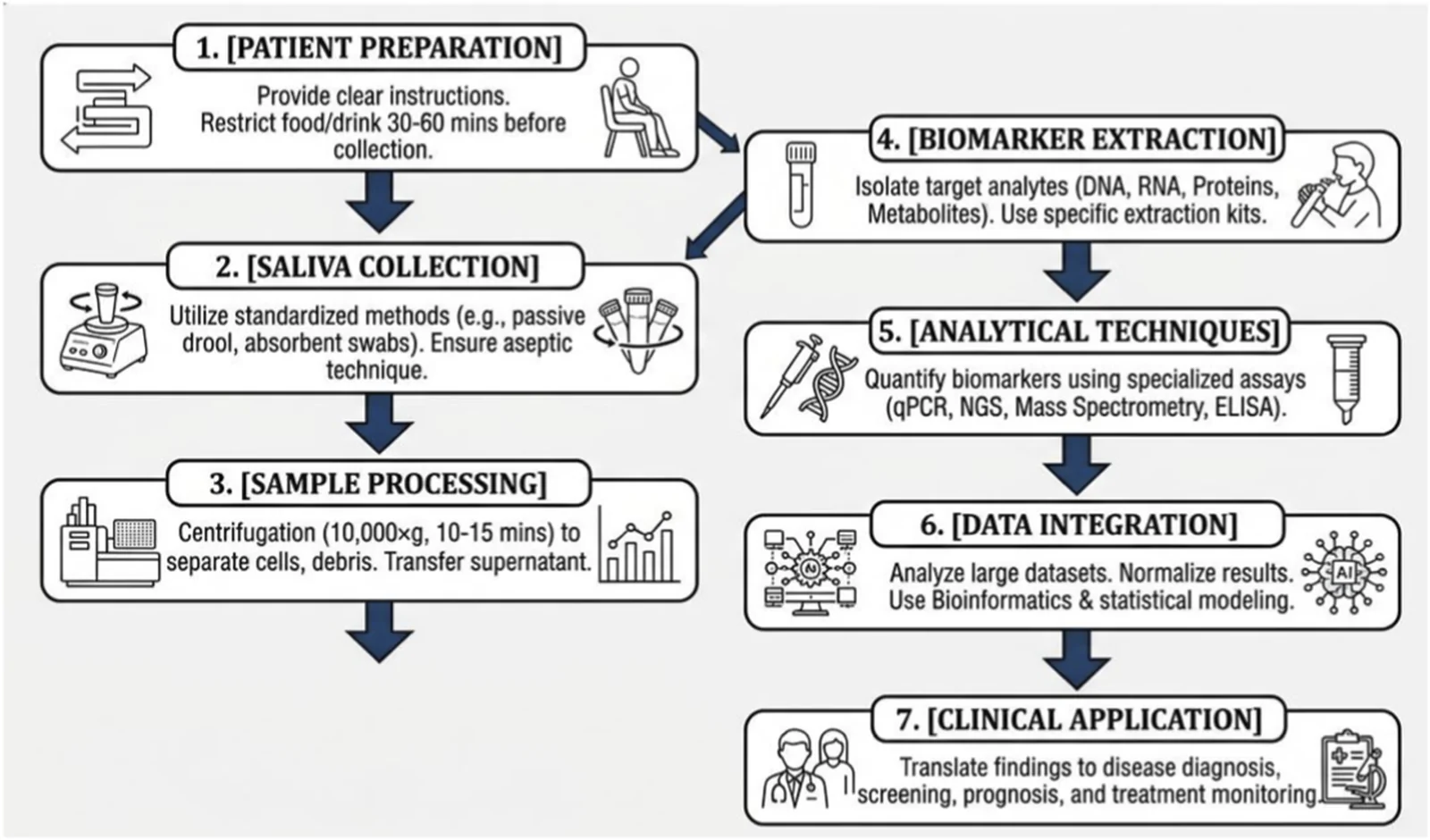
Workflow of salivary genomic biomarker analysis.

## 8 Multi-omic modeling, platforms, and sensors

### 8.1 Selecting the platform according to the intended use

To conduct triage and screening, assays must be fast, inexpensive, and deployable close to the point-of-care. RT-qPCR array and targeted methylation assays are viable options. To monitor this, special NGS or targeted assays can identify low-frequency variants and provide customized monitoring (Cui et al., 2021; Dal et al., 2024; Flach et al., 2022). Digital droplet PCR can help fill the gap between qPCR and NGS on prespecified targets.

### 8.2 Salivaomics and novel point-of-care sensing

The reviews of salivomics treat the diagnostics of saliva as a multilayered ecosystem comprising genomic, epigenomic, transcriptomic, proteomic, metabolomic, and microbiome characteristics. A 2023 review by the *Journal of the American Dental Association* (JADA) talked of salivaomics, saliva exosomics, and saliva liquid biopsy and mentioned electrochemical sensing methods to implement deployable diagnostics (Nonaka and Wong, 2023). Sensor platforms are attractive to scaling early detection, but, typically, such platforms must be reduced to a small number of highly informative targets. Thus, the development of biomarkers must explicitly consider downstream platform constraints at the early stages.

### 8.3 Machine learning: integration, validation, and transparency

In small datasets, machine learning is likely to overfit because multi-omic features, and clinical metadata can be combined into one risk score. Credible translation requires prespecified modeling plans, nested cross-validation, external validation cohorts, and calibration reporting. Longitudinal, within-person modeling surveillance promises to reduce inter-individual variability and detect molecular change earlier.

## 9 Intended-use scenario evidence

### 9.1 Screening of high-risk populations

The screening process must be balanced in both sensitivity and specificity because the downstream activity is biopsy. Screening of high-risk groups is most probably possible and more important than population-wide screening. Synthesis of evidence indicates inconsistency in study quality and reporting; many signatures are based on case–control studies enriched with cancers, which have the potential of overinflating performance (Khijmatgar et al., 2023).

A screening study ought to be translationally helpful by recruiting consecutive high-risk persons, taking saliva at predetermined intervals, and adjudicating the results against histopathology and follow-up studies. Notably, the comparison groups should be provided with inflammatory lesions and OPMDs to show the actual range observed in practice.

### 9.2 Triage of suspicious lesions

Triage is the imminent use. Saliva tests would be helpful to dental and primary care providers to prioritize referral and biopsy among patients with suspicious lesions. A triage test should be more valuable than normal examination and provide better time-to-referral or fewer unnecessary biopsies. Therefore, triage validation must recruit consecutive lesion-presenting patients (including inflammatory mimics) and compare the results of biomarker-assisted triage with the standard-of-care results.

The salivary autoantibody panels would present one such clinically available molecular method: a 2022 study in *Frontiers Oncology* created a salivary autoantibody panel to diagnose oral cavity SCC (Hsueh et al., 2022). Although autoantibodies do not imply genetic terms, this highlights why it is best to use multi-omic panels of orally based genomic and non-genomic markers.

### 9.3 Surveillance in case of treatment and minimal residual disease

The most competitive application of saliva genomics today is surveillance. The concept of tumor-informed mutation tracking is compatible with oncology follow-up regimes and has high specificity. The 2021 OSCC longitudinal study, as well as the 2024 pilot study of the *Frontiers Oncology* article, support the feasibility of detecting serial saliva mutations as a method of relapse monitoring (Cui et al., 2021; Dal et al., 2024).

To provide evidence of clinical utility, future studies should show changes in management (previously conducted imaging, targeted biopsy, and enhanced surveillance) based on the molecular results and the possibility that the same would lead to better outcomes or less treatment burden.

### 9.4 Risk of OPMD following and malignant transformation risk

OPMDs (e.g., leukoplakia, erythroplakia, oral submucous fibrosis, and lichen planus in some situations) are clinically significant because they present a useful population to detect and stratify with regard to risk. However, some patients cannot undergo repeated biopsies, and the clinical appearance does not consistently correlate with the molecular risk. Saliva-based signatures would help in risk stratification by the field effects, epigenetic dysregulation, and chronic inflammatory signaling. Nonetheless, in order to demonstrate value in predicting malignant transformation, testing should be longitudinal with an adequate follow-up period because the events of malignant transformation are comparatively rare and may take years.

One way that such testing can be implementation-friendly is to create a saliva risk score that would initiate an increase in clinical monitoring or biopsy on high-risk trajectories, as opposed to finding a single cross-sectional diagnostic label. This is a practice that has proven to work in other chronic disease surveillance and may help decrease unnecessary biopsies and detect high-risk patients earlier.

### 9.5 Complementary non-genomic molecular layers: what is their significance?

Even though this review dwells on genomic signatures, translationally it would be more plausible that joint panels would have superior performance. Multi-omic discussions and systematic reviews of oral cancer refer to the importance of proteomic and inflammatory mediator panels, which could be useful to supplement genomic characteristics in validated cohorts. An example is a 2021 *J Clin Med* article that compared leukoplakia and OSCC salivary cytokine panels; one reviewer highlighted the need to control periodontal and systemic inflammatory confounding, which is also pertinent to genomic testing (Dikova et al., 2021). Thus, the conceptual model of a genomic core of a larger saliva-based molecular risk model can be considered as the future genomic signatures.

## 10 Critical problems, prejudice, and adoption

### 10.1 Spectrum bias and the health control bias

One of the biggest challenges to translation is spectrum bias: much of the research uses established cancers as controls against healthy ones and poses a falsely easy classification task. Cohorts in the real world consist of inflammatory lesions, traumatic ulcers, infections, and OPMDs. The biomarkers that sound very precise in case–control designs lose their effectiveness in such natural cohorts. Cohort design and external validation in representative populations that are driven by the intention to use are needed.

### 10.2 Reproducibility and transfer in the laboratory

Genomic signatures are susceptible to batch effects that are contributed by extraction kits, sequencing chemistry, and bioinformatic pipelines. Shared controls, ring testing, and preferably centralized bioinformatics should be used in multi-site studies. Reducing signatures to a few stable features can enhance robustness and transfer ease to the clinical labs.

### 10.3 Pathways to regulation and clinical governance

Regulatory preparedness is based on intended use. A screening test is expected to have more evidence than a test used as an adjunct triage supporter. There is also the need to govern sample handling, reporting, and follow-up pathways in order to implement them clinically. Quality systems used in environments where laboratory-developed tests (LDTs) are made must be able to maintain a similar performance over time. In the case of sequencing-based assays, bioinformatics pipelines should be locked and version controlled, and performance should be documented across runs.

### 10.4 Economics of health, acceptability, and equity

Any program that is based on saliva must fall within real-world processes and be affordable. The collection of saliva is inexpensive; however, molecular assays may be expensive; the cost-effectiveness of the test may depend on its price, the prevalence of the population being tested, and the cost of the downstream procedures. Due to the higher burden of OSCC in underserved communities, implementation should be equity conscious: deployable guidelines on low-resource dental environments and close attention to the further increase of disparities are needed.

Patient acceptability must be high. False positives must be followed by counseling and clear referral pathways. False negatives are dangerous because they give false reassurance; hence, negative findings should never dominate clinical judgments in symptomatic lesions or in lesions with high suspicion.

### 10.5 Limitations of the study

This review has several limitations. First, much of the available evidence is derived from small-scale or case–control studies, which may introduce spectrum bias and overestimate diagnostic performance (Khijmatgar et al., 2023). Second, significant variability exists in saliva collection, processing, and analytical methodologies, limiting reproducibility across studies. Third, many studies are enriched with late-stage cancer cohorts, which may not accurately reflect early detection performance. Fourth, oral inflammatory conditions and microbiome variability can confound biomarker interpretation (Papale et al., 2022; Khijmatgar et al., 2023). Finally, there is a lack of large prospective multicenter validation studies, which is essential before clinical implementation.

## 11 Future directions and translational strategies

Future research should prioritize standardization of saliva collection, processing, and analytical workflows to improve reproducibility and reduce variability (Khijmatgar et al., 2023). Large-scale, multicenter prospective studies are essential to validate promising biomarkers across diverse populations. Integration of multi-omic approaches, including genomic, epigenomic, and transcriptomic data, with artificial intelligence and machine learning models holds significant potential to enhance diagnostic accuracy and clinical utility (Nassar et al., 2024; Wang et al., 2023). Furthermore, development of cost-effective, point-of-care diagnostic platforms will be critical for widespread adoption, particularly in low-resource settings (Nonaka and Wong, 2023). Successful translation will depend on bridging laboratory discoveries with clinical workflows, regulatory approval pathways, and health-economic feasibility.

## 12 Conclusion

During 2020–2024, saliva-based genomics for OSCC progressed from exploratory biomarker discovery toward clinically aligned applications, particularly in surveillance and triage. Longitudinal mutation tracking studies support feasibility for posttreatment monitoring, while systematic evidence synthesis highlights a growing but heterogeneous biomarker landscape. Clinical translation now depends on intended-use focused assay design, standardized pre-analytics, multicenter prospective validation, and demonstration of clinical utility with equity-aware implementation (Table 1).

**TABLE 1:** Saliva-based genomic/epigenomic biomarker classes relevant to OSCC (2020–2024 focus).

Biomarker class	Fraction	Representative platforms	Best-fit intended use	Strengths	Key limitations
Somatic mutations (ctDNA)	Cell-free saliva; oral rinse; pairedplasma	Tumor-informed NGS; ddPCR	Surveillance/MRD	High specificity; longitudinal tracking	Needs tumor tissue; low tumor fraction early
Personalized ctDNA (context)	Plasma ± saliva	Personalized deep sequencing	Surveillance/MRD	Actionable trajectories	Cost; logistics; assay standardization
DNA methylation panels	Cell-free saliva; cellular DNA	MSP; targeted bisulfite sequencing	Triage/risk stratification	Early event; stable signal	Bisulfite variability; inflammation confounding
mRNA signatures	Whole saliva; cellular fraction	RT-qPCR; targeted panels	Triage (adjunct)	Pathway-level information	RNA instability; batch effects
miRNA panels	Whole saliva; EV-enriched	RT-qPCR; small RNA-seq	Triage/screening (needs validation)	Stability and strong literature base	Normalization and population heterogeneity
EV nucleic acids	Isolated EVs	EV isolation + qPCR/NGS	Specialist triage/mechanistic profiling	Protected/enriched cargo	Isolation complexity and cost

## References

[B1] Birknerova N. Mancikova V. Paul E. D. Matyasovsky J. Cekan P. Palicka V. (2022). Circulating cell-free DNA-Based methylation pattern in saliva for early diagnosis of head and neck cancer. Cancers 14:4882. 10.3390/cancers14194882 36230805 PMC9563959

[B2] Bray F. Ferlay J. Siegel R. L. Laversanne M. Soerjomataram I. Jemal A. (2021). Global cancer statistics 2020: GLOBOCAN estimates of incidence and mortality worldwide for 36 cancers in 185 countries. CA Cancer J. Clin. 71:209–249. 10.3322/caac.21660 33538338

[B15] Chandrasekaran Y. Kayal L. Babu N. A. Kumar M. Jothinathan D. Behera A. (2024). Salivary biomarkers: a promising avenue for advancing oral cancer detection. Oral Oncol. Rep. 10, 100315. 10.1016/j.oor.2024.100315

[B3] Cui Y. Kim H. S. Cho E. S. Han D. Park J. A. Park J. Y. (2021). Longitudinal detection of somatic mutations in saliva and plasma for the surveillance of oral squamous cell carcinomas. PLoS ONE 16:e0256979. 10.1371/journal.pone.0256979 34478472 PMC8415592

[B4] Dal Secco C. Tel A. Allegri L. Baldan F. Curcio F. Sembronio S. (2024). Longitudinal detection of somatic mutations in the saliva of head and neck squamous cell carcinoma–affected patients: a pilot study. Front. Oncol. 14:1480302. 10.3389/fonc.2024.1480302 39555458 PMC11564150

[B5] Dikova V. Jantus-Lewintre E. Bagan J. (2021). Potential non-invasive biomarkers for early diagnosis of oral squamous cell carcinoma. J. Clin. Med. 10:1658. 10.3390/jcm10081658 PMC8070511 33924500

[B6] Flach S. Howarth K. Hackinger S. Pipinikas C. Ellis P. McLay K. (2022). Liquid BIOpsy for MiNimal RESidual DiSease detection in head and neck squamous cell carcinoma (LIONESS)—a personalised circulating tumour DNA analysis in head and neck squamous cell carcinoma. Br. J. Cancer 126:1186–1195. 10.1038/s41416-022-01716-7 35132238 PMC9023460

[B7] Hsueh P.-C. Chang K. P. Liu H. P. Chiang W. F. Chan X. Y. Hung C. M. (2022). Development of a salivary autoantibody biomarker panel for diagnosis of oral cavity squamous cell carcinoma. Front. Oncol. 12:968570. 10.3389/fonc.2022.968570 36387116 PMC9659860

[B8] Khijmatgar S. Yong J. Rübsamen N. Lorusso F. Rai F. Cenzato N. (2023). Salivary biomarkers for early detection of OSCC and HNSCC: a systematic review and network meta-analysis. Jpn. Dent. Sci. Rev. 60: 32–39. 10.1016/j.jdsr.2023.10.003 38204964 PMC10776379

[B9] Lingen M. W. Kalmar J. R. Karrison T. Speight P. M. (2008). Critical evaluation of diagnostic aids for the detection of oral cancer. Oral Oncol. 44:10–22. 10.1016/j.oraloncology.2007.06.011 17825602 PMC2424250

[B10] Liu D. Xin Z. Guo S. Li S. Cheng J. Jiang H. (2021). Blood and salivary MicroRNAs for diagnosis of oral squamous cell carcinoma: a systematic review and meta-analysis. J. Oral Maxillofac. Surg. 79:1082.e1–1082.e13. 10.1016/j.joms.2020.12.043 33516682

[B11] Nassar S. I. Suk A. Nguyen S. A. Adilbay D. Pang J. Nathan C. A. O. (2024). The role of ctDNA and liquid biopsy in the diagnosis and monitoring of head and neck cancer: towards precision medicine. Cancers 16:3129. 10.3390/cancers16183129 PMC11430155 39335101

[B12] Nguyen H. Nonaka T. (2024). Salivary miRNAs as auxiliary liquid biopsy biomarkers for diagnosis in patients with oropharyngeal squamous cell carcinoma: a systematic review and meta-analysis. Front. Genet. 15:1352838. 10.3389/fgene.2024.1352838 PMC10961377 38528913

[B13] Nonaka T. Wong D. T. W. (2023). Saliva diagnostics: salivaomics, saliva exosomics, and saliva liquid biopsy. J. Am. Dent. Assoc. 154:696–704. 10.1016/j.adaj.2023.05.006 PMC11390004 37500232

[B14] Papale F. Santonocito S. Polizzi A. Giudice A. L. Capodiferro S. Favia G. (2022). The new era of salivaomics in dentistry: frontiers and facts in diagnosis and management of oral disorders. Metabolites 12:638. 10.3390/metabo12070638 PMC9319392 35888762

[B16] Salivary Exosomes as Biomarkers for Early Diagnosis of Oral Squamous Cell Carcinoma. (2023). (PII: S2772906023000079).

[B17] Wang S. Yang M. Li R. Bai J. (2023). Current advances in noninvasive methods for the diagnosis of oral squamous cell carcinoma: a review. Eur. J. Med. Res. 28:53. 10.1186/s40001-022-00916-4 PMC9880940 36707844

[B18] Warnakulasuriya S. (2009). Global epidemiology of oral and oropharyngeal cancer. Oral Oncol. 45:309–316. 10.1016/j.oraloncology.2008.06.002 18804401

[B19] Yang R. Li T. Zhang S. Shui C. Ma H. Li C. (2024). The effect of circulating tumor DNA on the prognosis of patients with head and neck squamous cell carcinoma: a systematic review and meta-analysis. BMC Cancer 24:1434. 10.1186/s12885-024-13116-6 PMC11580464 39574043

